# Essential Tremors: A Literature Review of Current Therapeutics

**DOI:** 10.7759/cureus.59451

**Published:** 2024-05-01

**Authors:** Maurya D Patel, Muskaan Patel, Rutva Jani, Kishan G Patel, Priyansh Patel, Siddharth Kamal Gandhi

**Affiliations:** 1 Department of Internal Medicine, Smt. Nathiba Hargovandas Lakhmichand (NHL) Municipal Medical College, Ahmedabad, IND; 2 Department of Internal Medicine, Hinduhridaysamrat Balasaheb Thackeray Medical College (HBTMC) and Dr. Rustom Narsi Cooper Municipal General Hospital, Mumbai, IND; 3 Department of Internal Medicine, C.U. Shah Medical College and Hospital, Surendranagar, IND; 4 Department of Internal Medicine, B.J. Medical College, Ahmedabad, IND; 5 Department of Internal Medicine, Medical College Baroda, Vadodara, IND; 6 Department of Internal Medicine, M.P. Shah Government Medical College, Jamnagar, Jamnagar, IND

**Keywords:** focused ultrasound thalamotomy, deep brain stimulation, primidone, propranolol, essential tremor (et)

## Abstract

Essential tremors (ETs) commonly manifest as involuntary shaking of the hands that disrupt daily activities. These tremors involve the central motor network of the cerebellum, thalamus, and cortical networks, leading to different clinical phenotypes. The goal of this review was to establish evidence-based recommendations for effective care and simplify decisions for those dealing with ET. For this narrative literature review, we conducted a thorough search using core keywords such as “essential tremor” and “therapy.” From the 27 selected articles, relevant data were presented regarding pathophysiology, medications, and other treatment options, with necessary supplemental data such as side effects and use cases. This paper examines treatments for ET, including commonly prescribed medications such as propranolol and primidone; invasive treatments such as deep brain stimulation, focused ultrasound thalamotomy, transcranial magnetic stimulation, and some surgical methods; and non-invasive methods such as the neuromodulation technique of transcutaneous afferent patterned stimulation. Overall, this study presents a synthesized understanding of the currently available modalities for managing ETs. It is intended to guide care providers in choosing the best possible method to contain symptoms.

## Introduction and background

Essential tremor (ET) is the most common movement disorder, defined as an isolated tremor syndrome without other neurological signs, and presents with action tremor of the bilateral upper limbs for at least three years with or without involvement of the head, voice, or lower limbs [1]. ET stands out as a prevalent neurological movement disorder, exhibiting a prevalence rate of 3.06% among individuals aged 50 years or older. Typically, tremors associated with ET follow a progressive course, leading to eventual impairment in fundamental daily tasks, such as eating, writing, personal hygiene, and driving [2]. The origin of ET is not well understood, but it has been suggested that both genetic and environmental factors play a role. The specific mechanisms that contribute to ET are unknown, although research has shown that the gamma amino butyric acid (GABA) and glutamatergic systems, as well as the adrenergic and dopaminergic systems, and adenosine are involved [3]. The current treatments for ET include the use of non-selective β-blockers and anticonvulsants as first-line treatments, and topiramate, benzodiazepines, gabapentin, zonisamide, and pregabalin as second-line treatments. However, patients have variable responses to these medications [4]. Studies have shown that up to 74% of individuals with ET experience a significant reduction in tremor intensity after consuming small amounts of ethanol. However, ethanol is not a practical treatment option due to its narrow therapeutic window and the risk of excessive alcohol use [2]. Intramuscular injection of Botulinum toxin (BoNT) is an effective form of treatment for hand tremors and provides short-term relief without the risk of systemic adverse effects [5]. Prior studies have shown that applying electrical stimulation to peripheral nerves around the wrist can elicit activity within the ventralis intermedius (VIM) and other areas of the central tremor network. This has led to the creation of a non-invasive neuromodulation therapy, known as transcutaneous afferent patterned stimulation (TAPS) [4]. Transcranial direct current stimulation (tDCS) involves external stimulation of the cerebellum to reduce ET and has also been proven to be efficacious [6]. VIM-deep brain stimulation (VIM-DBS), radiofrequency thalamotomy, and magnetic resonance imaging-guided focused ultrasound (MRgFUS) thalamotomy are surgical techniques for treating ET. However, due to their invasive nature and potential for permanent side effects, these options are typically reserved as a last-line treatment for a select few patients [7]. The array of available treatments for ET presents a wide spectrum of results, affordability, side effects, and methods of application. This narrative review intends to thoroughly explore existing therapeutic options for ET, with the goal of providing well-supported advice for the selection of suitable treatments tailored to individual patient needs.

Methodology

We searched for literature across the PubMed, Medline, PubMed Central, and Google Scholar databases with the core keywords being “essential tremor” and “therapy.” The search query “essential tremor/therapy” [MeSH Major Topic] initially yielded 953 results. Studies that were not conducted on humans were omitted from the collection, along with studies that were not published in English. The remaining articles were screened for abstract and full-text availability, leading to a remaining total of 292 articles. Original articles were included according to their respective study designs, mainly clinical studies, observational studies, and randomized controlled trials for conclusive evidence. Case reports were excluded. Detailed eligibility criteria are shown in Table 1. After a careful analysis by the authors, 27 articles were included in this review.

**Table 1:** Eligibility criteria for the included studies. RCT: Randomized controlled trials

Inclusion Criteria	Exclusion Criteria
Papers in English language and available as free full text.	Papers in any other language than English language
Papers involving the use of keywords like essential tremors and therapeutics.	Studies with animal models.
Papers from all times	Papers that are not available as a free full-text version.
Papers with human participants are included	Gray literature.
Only RCTs, reviews, and observational studies are included.	Case reports, case series, and editorials are excluded.
Participants with all age groups are included	-

## Review

Pathophysiology

ETs arise from a central motor network, such as that controlling voluntary motor function. The cerebellum and thalamus play important roles in ET genesis. Cortical networks also contribute dynamically to establish a bilateral interaction between the cortex and the thalamus, which leads to involuntary tremors, as opposed to a unilateral interaction in physiologic voluntary movement [8]. The pathogenesis of ET has been explained by many theories. The central oscillatory network hypothesis is a better-accepted theory. This suggests that a network of oscillating neurons from different brain structures, namely, the inferior olive nuclei, cerebellum, thalamic motor nuclei, and motor cortex, collectively drive the tremor, as opposed to a single source. Neuroimaging studies have also revealed abnormal activation of these regions during tremors. Other proposed theories, such as the neurodegenerative hypothesis, suggest progressive Purkinje cell loss and cerebellar changes, and the GABAergic hypothesis implicating reduced GABAergic inhibition presents alternative evidence for ET pathology [9]. Holtbernd et al. provided a review of the imaging evidence of probable pathogenic mechanisms. Diffusion tensor imaging provides reliable proof of microstructural degeneration in the cerebellum, leading to altered function and circuitry. Cerebellar and thalamic GABAergic dysfunction in ET has been proven by radiotracer imaging studies. Epidemiological, genetic, and clinical heterogeneity with specific brain changes also play an important role in pathogenesis. Overall, ET is not a single entity but a spectrum with a clinical phenotype linked to specific brain changes [10].

Pharmacotherapy for ETs

The medical treatment of ET is based on the severity of symptoms, and medication is not given for mild cases. The onset of functional impairment prompts patients to seek treatment. Patients rely on alcohol consumption to temporarily dampen their symptoms; however, this is not a sustainable solution [11].

Propranolol

The first-line treatment is pharmacotherapy in the form of propranolol and primidone. Both were reiterated as Level A, effective in an updated guideline from 2011 [11]. Propranolol - a beta-adrenergic blocking agent, was one of the first drugs proven for the treatment of ET. Propranolol, at doses up to 360 milligram per day (mg/day), demonstrated up to 70% effectiveness in reducing limb tremors, as indicated by various measures of improvement. However, functional improvement and sustained treatment effects were less pronounced. Bradycardia and bronchospasm were the most common side effects, leading to discontinuation in less than 10% of the patients. Additionally, caution is advised when using propranolol in patients with asthma and chronic obstructive pulmonary disease, as it may worsen congestive heart failure [12]. In most countries, propranolol is recognized as a treatment for tremors. Approximately half of the patients experienced a significant decrease in the tremor intensity [13].

Primidone

Primidone, a first-generation barbiturate-type antiepileptic, is widely used in ET. It effectively reduces limb tremor severity and improves task performance and activities of daily living, with a higher patient preference compared to propranolol. However, primidone also causes frequent side effects, such as sedation, fatigue, nausea, and imbalance at doses from 150 to 750 mg/day, leading to discontinuation in up to 42% of patients. While primidone is equivalent to propranolol in terms of efficacy, its use requires specialized monitoring because of the risk of significant side effects that may limit its tolerability [12]. Primidone is indicated in patients who cannot tolerate or are resistant to propranolol. In young patients without comorbidities, propranolol was preferred, and in elderly patients with or without comorbidities, primidone was the preferred pharmacotherapy. If both are individually ineffective, then a combination of the two can be used before moving on to other non-pharmacologic therapies [13].

Topiramate

Topiramate is a more recently studied and proven alternative and is among the first-choice drugs for ET. As an anticonvulsant, its mechanism of action lies in agonism of the GABA-A receptor [14]. A meta-analysis conducted by Chang et al. demonstrated that topiramate (200 mg or 400 mg) was more effective than placebo in improving tremor symptoms. This was determined using the Fahn-Tolosa-Marin tremor rating scale (TRS) to measure changes. Upper limb tremor severity and functional disability were significantly reduced, and motor function was improved [15]. The latest guideline update stated that common adverse events (AEs) such as paresthesia, impaired concentration, suppression of appetite, and nausea were limiting in 31.9% of cases. Topiramate was proven to be clinically efficacious for upper limb tremors at doses up to 200 mg [12].

Other Anticonvulsants

In addition to primidone and topiramate, other anticonvulsants, such as carisbamate and gabapentin, are both in the investigational stage, with insufficient evidence supporting their efficacy. However, levetiracetam and pregabalin were not found to be efficacious. Progabide is unlikely to be efficacious and is unlikely to be useful [12].

Benzodiazepines

Alprazolam, a short-acting benzodiazepine, is an effective agent for the treatment of panic attacks and generalized anxiety disorders. A trial from Turkey demonstrated a 25% improvement in tremors, with an average daily dose of 0.75 mg. No other side effects were noted, except mild fatigue, sedation, and cognitive defects. Moreover, alprazolam is indicated, especially in elderly patients with intolerance to or contraindications to other agents [16].

Miscellaneous

Other drug categories such as calcium channel blockers (flunarzine and nimodipine), carbonic anhydrase inhibitors (methazolamide and acetazolamide), individual drugs such as amantadine, mirtazapine, olanzapine, and a new drug called “T2000” are also mentioned as possible treatments [13].

Botulinum Toxin (BoNT)

BoNT type A (BoNT-A) intramuscular injections are indicated for disabling upper limb and wrist tremors as a form of targeted local therapy if oral formulations are ineffective. The locations of muscle injections are chosen after careful kinematic analysis of limb movement so that the side effects - wrist weakness and reduction in grip strength - are minimized [16,17]. Furthermore, a recent trial showed the efficacy of BoNT-A injections in reducing the severity of isolated head tremors after 18 weeks of therapy but resulted in side effects such as neck muscle pain, weakness, and dysphagia [18].

Octanoic Acid

**Figure 1:**
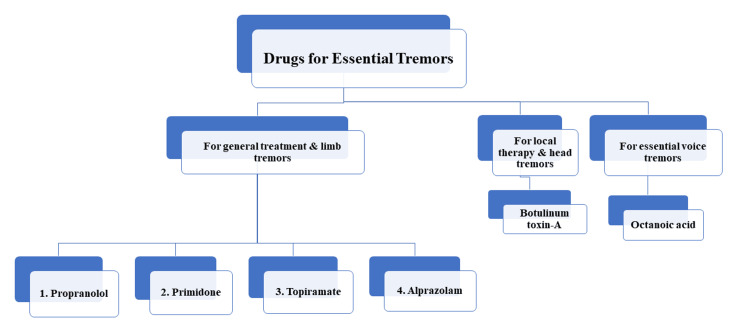
Pharmacotherapy of essential tremors

Another, less addressed, manifestation of ET is voice tremors, where the patient has speech issues due to voice shakiness and hoarseness. For these essential voice tremors (EVT), octanoic acid is a primary option. A dose of 200 mg or 400 mg for three weeks was effective in reducing the severity of voice tremors affecting the oropharyngeal and laryngeal regions in one trial, but there is a lack of further meaningful clinical evidence [19]. A flowchart representing the pharmacotherapy of ETs is shown in Figure 1.

Invasive therapies

Medication refractory patients are those who have lasting disabling effects of tremor regardless of no less than two full-dose trials of approved therapeutic agents, one of which has to be propranolol or primidone. In this fraction, invasive methods are the last resort when noninvasive methods have shown no benefit [20].

Deep Brain Stimulation (DBS)

Until the late 19th century, thalamotomy was the mainstay of surgical intervention and later replaced by DBS [21]. DBS is known to modify the pathologic activity within the cerebello-thalamo-cortical connections, that is, the dentato-rubro-thalamic tract (DRTT), which is known to constitute the tremor network [22]. Once targets are identified by preoperative axial magnetic resonance imaging (MRI) sequences and stereotactic computed tomography (CT) scans, electrodes with four to eight contacts are placed in the burr holes created in the cranium, only after clinical stimulation tests are performed to define tremor suppression threshold and adverse effects. The pulse generator lodged in the subclavicular region is connected to the electrodes through extensions. The strength of the generated pulse has a direct relationship with the current flowing through the contacts and radius of the functional tract blockade [13,21,23]. VIM-DBS has emerged as a potential treatment for ET with a better response and outcomes than thalamotomy [23]. Results of a retrospective cohort involving 28 patients undergoing DBS showed persistent reduction in tremor even at 10 years post-surgery, although with gradually decreasing efficacy, 66% improvement at one year follow, and 48% improvement at 10 years follow-up as compared to baseline [21]. As pointed out by various studies, the posterior subthalamic nucleus (PSA), which also encompasses the caudal zona incerta, promises to be a better stereotactic target [22]. In a Norwegian study, nine out of 10 patients showed sustained effective tremor suppression with PSA-DBS at one-year blinded follow-up [23]. Situated below the thalamic VIM, PSA is bounded anterolaterally by the subthalamic nucleus (STN) and medially by the red nucleus (RN) [23]. In their randomized controlled trial, Dembek et al. concluded that PSA stimulation led to greater tremor suppression than VIM stimulation at equal amplitudes [22]. This study revealed that the efficacy of a target lies in its proximity to DRTT [22]. Equivalent results may be achieved by stimulating a target farther away from the DRTT with a greater amplitude. However, this supratherapeutic stimulation has to be weighed against negative effects, such as dysarthria and paresthesia [23]. Gait ataxia is believed to occur because of the activation of retrograde fibers directed to the cerebellum [13]. Candidate selection is critical for successful DBS outcomes [24]. High cost and fitness for surgery were the defining factors for selection. Despite its therapeutic superiority, DBS is associated with complications, such as hemorrhage, paresthesia, gait disturbances, and dysarthria [23,25]. DBS surgery has a mean morbidity rate of 3.7%, which is mainly attributable to the most common complication, intracranial hemorrhage [13]. The most common cause of unintended hospitalization in these patients is a fall [24]. Interdisciplinary screening processes to minimize adverse effects have been widely accepted. It includes neurologists, neurosurgeons, neuropsychologists, psychiatrists, and physical and occupational therapists [24]. Owing to the prevalence of mood disorders in these patients, psychiatrists might play a significant role in minimizing the frequency of falls in addition to physical therapists [24]. Despite its adverse effects, DBS is still considered the best and only invasive therapy in many locations worldwide. Its outstanding tremor improvement outcomes, along with the sustenance of effects in addition to cost-effectiveness, rightly outweigh the complications. 

Focused Ultrasound Thalamotomy

**Figure 2:**
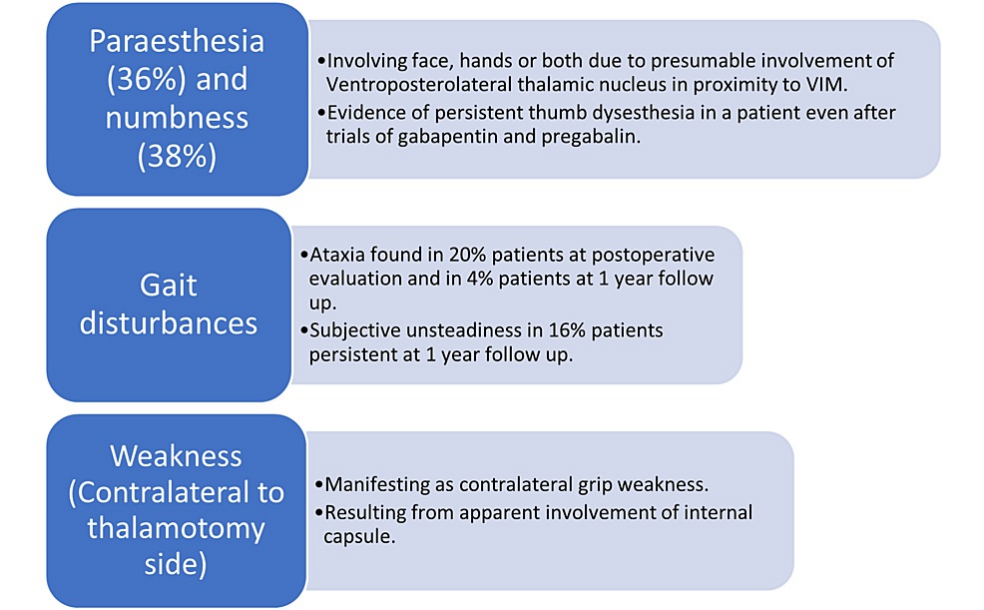
Side-effect profile of magnetic resonance imaging-guided focused ultrasound sonication

The beginning of the 20th century witnessed the advent of new ultrasound transducers capable of delivering focused energy through the intact skull. This along with continuous developments in the field of MRI have permitted millimeter-level accuracy of surgical planning and real-time non-invasive temperature monitoring [26]. MRgFUS is the result of these advances, wherein high-intensity focused ultrasonography creates a lesion by heating and ablating the tissues under MRI guidance throughout the intervention [26]. The patient’s fully shaved head was fixed in a stereotactic frame compatible with MRI, which was then positioned into the helmet-like cavity of the sonography transducers [26]. An elastic diaphragm filled with degassed water at a temperature of 16 °C filled the space between the head and transducer to ensure scalp cooling [20,26]. A series of magnetic resonance images fused with preoperative CT helps fix the target (VIM), keeping the anterior and posterior commissure line as a reference [26]. After target selection, low-power sonication that induces temperatures of less than 45 °is delivered for 10-20 seconds. Although such subthreshold temperatures produce reversible effects and are unlikely to cause lesioning, they are easily picked up on MR thermometry, which facilitates target confirmation and the prevention of deleterious permanent side effects [26]. The temperature gradually increased sequentially. The final sonication temperature ranged from 55 to 63 °C [20]. Since patients are awake throughout the procedure, clinicians freely communicate with them, enabling them to understand the improvement in tremor intensity, assess neurologic integrity, and observe any side effects such as paresthesia [20,26]. As evident on T2 weighted imaging and FLAIR, the lesion created by sonication shows three zones, zone one (hypointense central area of coagulation necrosis), zone two (strongly hyperintense zone demarcated by the hypointense rim, consistent with cytotoxic edema) and zone three (fuzzy, hyperintense zone of vasogenic edema) [20,26]. Several trials of MRgFUS have shown a significant reduction in tremor intensity and improvement in quality of life [27]. Remarkable tremor reduction at three months post-MRgFUS and its persistence at one-year follow-up were seen in a randomized control trial involving 76 drug-refractory patients [27]. A pilot study conducted in Virginia showed noteworthy tremor improvement in the hand contralateral to the lesion side, with a relative reduction of 75% from baseline and improvement in total tremor scores with a relative reduction of 56% as found on the one-year follow-up post-procedure, as measured by the clinical TRS [20]. Shankar et al. suggested in their clinical review that patients in the interventional group showed an improvement of 47% as compared to only 0.1% in the sham procedure group at three-month follow-up [21]. However, the benefits observed at two years post-procedure were of declining efficacy. Prospective causes of this multifactorial effect include reduced lesion size and diminishing perilesional edema [21]. One of the prerequisites for undergoing MRgFUS is a skull density ratio of 0.35-0.4 [13]. The risk of adverse effects increased with lesions measuring 170 cubic millimeters or more [21]. Although less invasive than DBS, MRgFUS has its own set of side effects, as highlighted by Elias et al. [20,21,27]. A detailed side-effect profile of MRgFUS is shown in Figure 2.

Nevertheless, MRgFUS is an excellent intervention in patients who are not considered fit for surgery or those with associated comorbidities, such as hypertension, who may suffer intracranial hemorrhage with an increased probability when undergoing DBS. In addition, lead revision or annual hardware removal pertaining to its programming or complications, such as infection, are eradicated in the case of MRgFUS.

Diffuse Tensor Imaging of Fiber Tracts (DTI)

DTI is a technique wherein individual axonal fiber tracts can be visualized using Diffuse Tensor Imaging, a type of MR imaging. This facilitates the selection of appropriate targets for invasive therapeutic techniques [28]. This not only reduces surgical time but also complications by lesioning or placing electrodes away from important tracts such as the pyramidal and spinothalamic tracts [28,29]. In a retrospective study by Sasada et al., four fiber tracts were studied; cerebello-thalamo-premotor cortical fiber tract (C-T-preM), cereblo-thalamo-primary motor cortical tract (C-T-M1), spino-thalamosensory cortical fiber tract (Sp-T-S1), and pyramidal tract (Py) [28]. As picked up on T1 and T2 weighted images, thalamotomy lesions were placed on C-T-preM and DBS electrodes were located within C-T-preM or on the border between C-T-preM and C-T-M1 [28]. Several studies have demonstrated the placement of effective DBS electrodes in the DRTT. There was a high mean diffusivity of cerebellar grey matter, as evidenced by a retrospective study involving 67 patients with ET, suggesting the presence of microstructural cerebellar changes [28]. There were no permanent adverse effects such as hemorrhage or ischemia. Transient contralateral lower-extremity hypotonia and dysarthria were observed in thalamotomy patients, and transient paresthesia in DBS patients disappeared within a few months [28].

Repetitive Transcranial Magnetic Stimulation (rTMS)

rTMS is a non-invasive brain stimulation (NIBS) therapy [30]. In this way, stimulating electrodes placed strategically near the patient’s scalp create a magnetic field by producing small electrical currents. The phenomenon of cerebellar inhibition has been known for years, wherein the magnetic stimulus applied to the cerebellum causes inhibition of the contralateral primary cortex through cerebello-thalamo-cortical (CTC) connections. This inhibition is caused by the use of low-frequency currents (less than one Hz), in contrast to high-frequency currents (five Hz and more), which cause cortical excitation [30]. On either side, coils are stationed tangentially to the cranial surface at a point distanced one-third from the inion on an imaginary line joining the inion and mastoid process. With respect to the midline, coils are given an angulation of 45° with the current flowing in the downward direction [30,31]. Fahn Tolosa Marin clinical scale (FTM), having a total point of 156, has been widely used to evaluate tremor effects. It includes three subscales; Part A (tremor severity/amplitude), Part B (specific motor task assessment such as writing, spiral drawing, and pouring water with both hands), and Part C (functional disability measurement in activities of daily living) [30]. In a randomized controlled trial conducted in China, patients receiving rTMS therapy were given a total of 600 pulses, 20 pulses for 30 seconds followed by 5 second break. This study demonstrated improvement in FTM scores including parts A and B only after being subjected to 20-minute rTMS sessions for 10 days, with effects lasting up to 20 days [30]. This indicated that tremor severity was reduced without significant improvement in functional disability in daily living. This study also mentioned that the improvement was statistically the same as that in patients on propranolol therapy. Lv et al. showed considerable improvement in all the subscales of FTM, with effects lasting up to three weeks after the last session [30]. Alternatively, there have been studies showing no improvement in tremors. In a double-blind sham-controlled trial conducted by Olfati et al., patients in the rTMS group received 900 pulses daily for five days. No significant tremor improvement was observed at any time point. However, a placebo effect with 70% improvement was observed in a patient in the sham procedure group [31]. Adverse effects reported are headache, local pain, visual disturbances, neck spasms, and lightheadedness, all transient in nature and disappear after a few hours [31]. rTMS is a therapy that can be considered in patients who do not want to undergo surgical intervention and are accepting with regard to the persistence of disabling tremor effects [13].

Radiofrequency Lesioning

Over the past 100 years, many patients have undergone radiofrequency lesioning of the well-known target, VIM. It is a neurosurgical procedure wherein special electrodes capable of producing high-intensity current are placed in the target area once identified on preoperative CT or MRI. The current flowing through the electrodes produces temperatures as high as 60 °C, which leads to local tissue ablation. The electrodes were then removed [13]. Proper tract selection and target confirmation are facilitated by the use of microelectrodes. Patients were awake throughout the procedure. Few studies have shown an improvement in lateralized outcomes by approximately 74% from baseline [13]. Pertaining to its irreversibility, radiofrequency lesioning has been associated with a higher number of side effects. Permanent speech disorders with bilateral lesions have been reported more frequently. Owing to its high AE rate and the advent of better surgical techniques, this procedure has been on the brink of disuse [13].

Radiosurgery

It is a rare procedure performed in a radiation suite and is restricted to a limited number of highly specialized centers. Focal radiation destroys the target area (VIM) obtained from imaging studies. The principle underlying the development of effects as well as AEs is based on the occurrence of post-radiation tissue reactions in the form of scarring [24]. Three studies demonstrated an improvement of 55.9% on the TRS with sustenance for up to four years. A meta-analysis reported that the adverse effects were as low as 0.7%. However, radiosurgery lacks reliability owing to the presence of only a few studies [24].

Non-invasive therapy

The results of the study indicate that using non-invasive neuromodulation therapy tailored to the individual at home for more than three months can lead to a reduction in hand tremors and an improvement in the quality of life for many patients with ET [4].

Transcutaneous Afferent Patterned Stimulation (TAPS)

TAPS is a wrist-worn, non-invasive therapy that delivers calibrated stimulation to the median and radial nerves [25]. TAPS is a therapy that involves non-invasive electrical stimulation of the median and radial nerves at the wrist, with a frequency tailored to each patient's tremor. Two randomized, single-session studies with sham control have shown that TAPS is a safe and effective treatment for symptomatic ET, leading to its clearance by the United States Food and Drug Administration (US-FDA) [4]. The treatment has been found to reduce tremors and has no serious device-related AEs, making it a safe and effective option for ET. Over 50% of patients experienced a ≥2-fold reduction in tremor power with daily TAPS therapy, and for most patients (64%), tremor relief lasted for an average of more than 90 minutes after the therapy session. These tremor reductions are comparable to those achieved with first-line pharmacotherapies, propranolol and primidone [4]. A large-scale pragmatic clinical trial conducted by Dai et al. evaluated unsupervised TAPS use in a real-world home-based setting, and the study demonstrated that adding TAPS therapy to the existing standard of care (SOC) reduced tremor power and improved upper limb scores on the Bergmann Fugl-Meyer Assessment (BFMA) during one month of home use compared to SOC alone. These findings support and expand upon previous prospective and real-world studies, suggesting that TAPS is a safe and effective treatment for patients with ET [25]. Pahwa et al. conducted a randomized controlled trial that compared the effects of noninvasive peripheral nerve stimulation to a sham group in patients with ET. The treatment group showed significantly greater improvements in functional outcome measures, such as the tremor research group essential tremors rating scale (TETRAS) upper limb tremor scores and activities of daily living (ADLs), as well as patient-reported outcomes, such as the clinical global impression-improvement (CGI-I) scores. These improvements corresponded to a 49% reduction in tremor according to ADLs and a 42% reduction according to TETRAS upper limb tremor following a single stimulation session. These results are clinically meaningful and fall within the range of tremor amplitude improvement reported in studies of medications commonly used to treat ET. Additionally, 75% of subjects had a response greater than 30% improvement, and 65% of subjects had a response greater than 40% improvement in TETRAS following a single stimulation session. Furthermore, 70% of subjects reported a greater than 30% improvement in ADLs, and 65% of subjects reported a greater than 40% improvement. These findings suggest that TAPS is a safe, effective, and patient-friendly noninvasive therapy for ET [32]. A pilot study by Lin et al. also found that noninvasive neuroperipheral therapy may offer clinically meaningful symptomatic relief from hand tremors in ET with a favorable side effect profile compared to other available therapies [33]. The studies highlighted in this review collectively demonstrate that TAPS is a safe, effective, and patient-friendly non-invasive therapy for ET. It not only reduces hand tremors but also improves daily functioning and quality of life, presenting a viable alternative to traditional pharmacotherapies. These findings suggest that TAPS could be a significant advancement in the therapeutic approach for ET.

Limitations

First, the review was confined to articles published in English, potentially excluding relevant studies published in other languages. Additionally, only articles with free full-text availability were considered, which may have led to the exclusion of pertinent research on paywalls. The literature search was limited to PubMed and PubMed Central databases, omitting studies that might be available in other databases. The scope of this review was restricted to studies involving human subjects, thereby excluding research on other species that could offer additional insights. A notable limitation is the inclusion of studies with shorter durations, which may have impacted the long-term applicability of the findings. Some studies included in the review had small sample sizes, potentially affecting the robustness and generalizability of the results. Finally, the review only considered articles published in the last decade, which might have overlooked relevant historical research or long-term trends in the field. Non-invasive therapies for ET, such as TAPS, have variable efficacy among individuals and often provide only temporary relief. They may be less effective in severe cases and lack precision targeting of invasive methods, underscoring the need for personalized and potentially more advanced treatment approaches.

## Conclusions

The article discusses the various management options available for ET, a neurological disorder that causes involuntary, rhythmic shaking of limbs and other body parts during voluntary movement. The primary approach to symptomatic management of ET has primarily been pharmacological, with propranolol and primidone being the first-line treatment options. Topiramate is also an effective treatment option when propranolol and primidone do not result in recovery. BoNT provides a local approach to short-term relief from hand tremors. In addition to drug therapy, surgical interventions such as focused ultrasound thalamotomy, radiofrequency ablation thalamotomy, and DBS can provide short-term relief; however, these techniques carry an increased risk of adverse surgical and neurological effects. Non-invasive techniques such as peripheral nerve stimulation have emerged as a viable alternative to invasive methods due to their increased safety and reduced risk of adverse effects, although they require newer infrastructure and training. According to the literature, functionally limiting symptoms are initially treated with drugs, and caregivers' resort to invasive and non-invasive methods if pharmacotherapy alone does not provide relief. This study provides a comprehensive overview of the management options available for different subtypes of ET, encompassing various modes of intervention. By providing information about specific indications and potential adverse effects for each therapeutic approach, healthcare professionals are equipped with essential knowledge to make informed decisions tailored to individual patient needs. Various ongoing trials of newer drugs have demonstrated their efficacy in treating ET, but there is a lack of evidence showing the long-term efficacy of widely used drugs and other methods in different kinds of ETs. More experiments on how diverse populations respond to different existing as well as newly tried drugs and methods could prove beneficial in developing a consensus for ET therapy.
